# Peripheral Inflammatory Markers Contributing to Comorbidities in Autism

**DOI:** 10.3390/bs6040029

**Published:** 2016-12-14

**Authors:** Martha Cecilia Inga Jácome, Lilia Maria Morales Chacòn, Hector Vera Cuesta, Carlos Maragoto Rizo, Mabel Whilby Santiesteban, Lesyanis Ramos Hernandez, Elena Noris García, Maria Elena González Fraguela, Caridad Ivette Fernandez Verdecia, Yamilé Vegas Hurtado, Dario Siniscalco, Carlos Alberto Gonçalves, Maria de los Angeles Robinson-Agramonte

**Affiliations:** 1 Immunology Department, Havana University Medical Science, Havana 11300, Cuba; martha@cngen.sld.cu or marthytuamiga@yahoo.com; 2 Neurophysiology Department, International Center for Neurological Restoration, Ave 25 # 15805 b/w 158 and 160, Playa, Havana 11300, Cuba; lily@neuro.ciren.cu (L.M.M.C.); marie@neuro.ciren.cu (M.E.G.F.); 3 Infant Neurology Clinic, International Center for Neurological Restoration, Ave 25 # 15805 b/w 158 and 160, Playa, Havana 11300, Cuba; verac@neuro.ciren.cu (H.V.C.); maragoto@neuro.ciren.cu (C.M.R.); 4 Psychiatry Department, Children Hospital Las Católicas, Calzada del Cerro entre Santa Teresa Y Monasterio, Havana 10200, Cuba; mabel.whilby@infomed.sld.cu (M.W.S.); leya@infomed.sld.cu (L.R.H.); 5 Immunology Department, Hospital Clinico Quirúrgico Docente “Joaquin Albarrán”, Havana 10600, Cuba; anoris@infomed.sld.cu; 6 Department of Experimental Model, International Center for Neurological Restoration, Ave 25 # 15805 b/w 158 and 160, Playa, Havana 11300, Cuba; ivettef@neuro.ciren.cu (C.I.F.V.); yvegas@neuro.ciren.cu (Y.V.H.); 7 Department of Experimental Medicine, University of Campania “Luigi Vanvitelli”, via S. Maria di Costantinopoli 16, Naples 80138, Italy; taodar@yahoo.it; 8 Departamento de Bioquímica, Instituto de Ciências Básicas da Saúde, Universidade Federal do Rio Grande do Sul, Porto Alegre 90040-060, Brazil; casg@ufrgs.br; 9 Neuroimmunology Laboratory, Immunochemical Department, Technology and Science Division, International Center for Neurological Restoration, Ave 25 # 15805 b/w 158 and 160, Playa, Havana 11300, Cuba

**Keywords:** Autism spectrum disorders, comorbidities, EEG, behavior, epilepsy, neuro-inflammation, social interaction

## Abstract

This study evaluates the contribution of peripheral biomarkers to comorbidities and clinical findings in autism. Seventeen autistic children and age-matched typically developing (AMTD), between three to nine years old were evaluated. The diagnostic followed the Diagnostic and Statistical Manual of Mental Disorders 4th Edition (DMS-IV) and the Childhood Autism Rating Scale (CARS) was applied to classify the severity. Cytokine profile was evaluated in plasma using a sandwich type ELISA. Paraclinical events included electroencephalography (EEG) record. Statistical analysis was done to explore significant differences in cytokine profile between autism and AMTD groups and respect clinical and paraclinical parameters. Significant differences were found to IL-1β, IL-6, IL-17, IL-12p40, and IL-12p70 cytokines in individuals with autism compared with AMTD (*p* < 0.05). All autistic patients showed interictalepileptiform activity at EEG, however, only 37.5% suffered epilepsy. There was not a regional focalization of the abnormalities that were detectable with EEG in autistic patients with history of epilepsy. A higher IL-6 level was observed in patients without history of epilepsy with interictalepileptiform activity in the frontal brain region, *p* < 0.05. In conclusion, peripheral inflammatory markers might be useful as potential biomarkers to predict comorbidities in autism as well as reinforce and aid informed decision-making related to EEG findings in children with Autism spectrum disorders (ASD).

## 1. Introduction

Autism spectrum disorders (ASD) are neurodevelopmental disorders defined by significantly abnormal social interaction, impaired communication and language difficulties, and narrow patterns of interests. Symptoms appear during infancy or childhood and generally are followed by a steady course without remission. Symptoms gradually begin after the age of six months to become established by age two or three years old and continue through adulthood, although generally more attenuated.

Growing data suggest the impact of immune dysfunction on neuron development and autism outcome [1,2,3,4,5,6,7,8]. Changes in cytokine levels have been reported in cerebrospinal fluid (CSF) of patients with autism, showing brain inflammation [6,7]. Previous reports on autism include findings regarding changes in IL-1β, IL-10, IL-6, IL-17, and IL-12 cytokines and differential cellular immune response associated with disease severity and impairment in behaviors in ASD [8,9,10]. In some cases, evidence has also been provided regarding cytokine’s role in mediating suppression of both FoxP3 (+) and FoxP3 (−) T regulatory cells in this disorder [11].

Evidence from other studies show an increased prevalence of epilepsy and/or abnormalities in electroencephalography(EEG) in these children, as well as an increased risk of seizures at puberty [12,13,14,15,16]. It is estimated that approximately one-third of children and adolescents with autism experiences seizures [17,18], nevertheless the role of inflammation in autism and its contribution to associated comorbidities, like epilepsy, continues to be a still unresolved question.

Evidence from several previous studies had marked the role of pro-inflammatory cytokines, such as IL-1, IL-6, and TNFα, as main factors in the generation of abnormal behavioral and electroencephalography patterns occurring in autism.The current study was designed to explore changes in cytokine profile in autism and their relevance to clinical and paraclinical parameters. This study evaluated the contribution of inflammation to clinical outcome and comorbidities in autism, showing that peripheral inflammatory markers might be used to predict comorbidities in autism as well as became useful to reinforce and aid informed decision-making related to EEG findings in these affected children.

## 2. Materials and Methods

### 2.1. Study Subjects

The study considered seventeen children with clinical diagnosis of ASD and fifteen age-matched typically developing (AMTD) children. The diagnosis was followed according to DSM-IV-TR 2000: the Diagnostic and Statistical Manual of Mental Disorders, Fourth Revision [19,20,21,22]. The autistic patient group was between three and nine years old, including 12 male and 5 female patients. The patients were also classified according disease severity. The Childhood Autism Rating Scale (CARS) test was used by research reliable severity score [23,24]: 1 patient who has a CARS score of 37 or more was classified as severe; 11 patients were classified with moderate severity having a CARS score between 32 and 37, and 5 patients were classified as mild, on account of their CARS score being below 32. Informed consent was obtained from parents or caregivers of children to be included in the study. The parents were also informed about the objective and benefits of the study as well as their right to be excluded at any time, under their decision. The study was carried out in accordance with the Helsinki norms and rights for human research and was approved by the Ethical Committee of the International Center for Neurological Restoration. The clinical characteristics of all participants are summarized in Table 1.

### 2.2. EEG Assessments

Routine awake and sleep 60-minute-EEG or both were performed with a Medicid-5 digital EEG system (Neuronic SA, Cuba), with 32 channels. Monopolar leads were employed, using Fpz electrodes as reference. Moreover, electrodes placed over the superior and inferior rim were used in order to record eye movement artifacts for easing their detection in the EEG records. EEG technical parameters were: gain 20,000, pass-band filters 0.1–70 Hz, “notch” filter at 60 Hz, noise level of 2 lV(root mean square), sampling frequency 200 Hz, and electrode–skin impedance never higher than 5 KX. EEG analyses were performed by two board-certified electroencephalographers.

Type and localization of EEG epileptiform abnormalities in awake/sleep-EEG recordings were evaluated. EEG studies were classified as epileptiform if spikes, sharp waves, or spike/slow waves complexes were recorded. Abnormalities such as slowing of the background or other non-epileptic pattern were not considered. All activity regarded as artefactual was excluded.

### 2.3. Cytokine Profile Measurement

#### 2.3.1. Samples Collection

Peripheral blood samples were collected from each subject for serum analysis. Whole blood was collected into ethylenediaminetetraacetic acid (EDTA) coated collection tubes. The tube was inverted 10 times to mix the blood with the EDTA, and stored on ice until further processing. In all cases, the duration from collection to freezing was noted. Blood was separated by centrifugation at 1000× *g* in a swinging bucket centrifuge pre-chilled to 4 °C. The plasma was harvested, placed into 200 μL aliquots, and stored at −80 °C until cytokine analysis (no freeze-thaw cycles occurred before analysis).

#### 2.3.2. Cytokine Analysis

Cytokine analysis was conducted using an array-based multiplex sandwich ELISA System (Quantibody^®^ Human Inflammation Arrays; QAH-INF-3, RayBiotech, Inc., Norcross, GA, USA) for the simultaneous quantification of multiple cytokines/chemokines: Interleukin (IL)1α, IL1β, IL1 receptor antagonist(IL-1ra), IL-2, IL-4, IL-5, IL-6 and its soluble receptor (IL-6sR), IL-7, IL-8, IL-10, IL-12p40, IL-12p70, IL-13, IL-15, IL-16, IL-17, eotaxin, eotaxin-2, granulocyte colony-stimulating factor (G-CSF), macrophage colony-stimulating factor (GM-CSF), Interferon (IFN)γ, monocyte chemotactic protein (MCP)-1, macrophage inflammatory protein (MIP)-1α, MIP-1β, MIP-1γ, platelet-derived growth factor (PDGF)-BB, regulated on activation normal t cell expressed and secreted (RANTES), tumor necrosis factor (TNF)-α, TNF-β, soluble receptors TNF-sRI, TNF-sRII, macrophage colony-stimulating factor (M-CSF), B-lymphocyte chemo attractant (CXCL13), I-309, intercellular adhesion molecule (ICAM)-1, monokine induced by gamma interferon (MIG), tissue inhibitor of metalloproteinase (TIMP)-1, TIMP-2.

Samples were evaluated in accordance with the instructions of the manufacturer. Briefly, one array was run to optimize the serum dilutions at which the cytokines would be quantified within the array’s limit of detection. All plasma samples and standards were incubated on the arrays at 4 °C and stored overnight with gentle shaking. After several washes, the detection antibody cocktail of Streptavidin-enzyme horseradish peroxidase conjugated was added, and the arrays were incubated at room temperature. After another series of washes and incubations, the reaction was stopped and the cytokine concentration was calculated. The data were expressed in pg/mL.

### 2.4. Data Analysis

Statistical analysis was performed using the Statistic 6 version Software. Categorical data were described by absolute frequencies and percentages and the continuous variables as median and standard error. The Kolmogorov-Smirnov and Shapiro-Willkie tests of normality were applied to analyze the normal distribution of the variables.Differences between groups and in respect to disease severity were determined by Student independent *t* tests. The associations between qualitative variables were evaluated by Fisher exact tests. Statistical significance was defined as *p* < 0.05.

## 3. Results

### 3.1. Clinical and Demography Description of the Group

The demography and clinical characteristics of autistic patients are summarized in Table 1. The total number was grouped to minimize comorbidities and other factors susceptible to introduce controversial results. Autistic patients (12 male/5 female; mean age 6.17 ± 2.08) and 15 age control subjects (10 male/7 female; mean age 6.5 ± 1.65) were evaluated. The disease severity was distributed as follow: 64.7% classified as moderate, 29.4% classified as mild, and 5.88% classified as severe.

### 3.2. EEG Assessment Records in Autistic Patients

**Figure 1:**
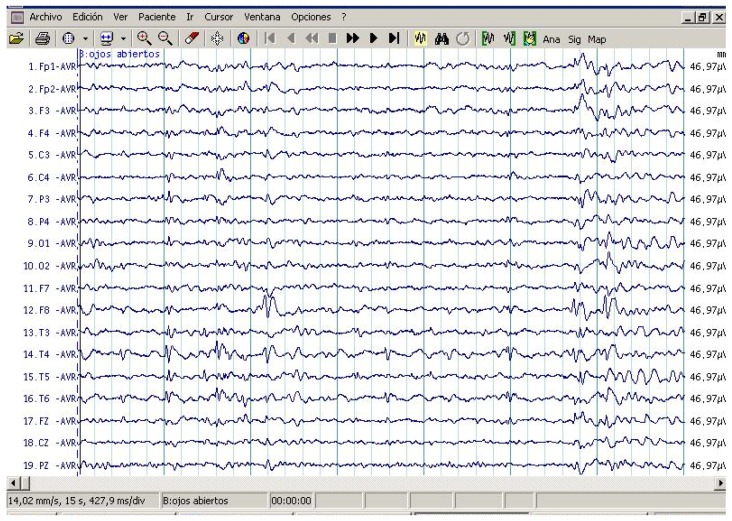
Routine awake electroencephalography (EEG) study in patient with ASD with epileptic seizures. Note EEG independently epileptiform abnormality in channels containing the frontal and temporal leads in both hemispheres. Maximum amplitude occurred in F8, T4, Fp1, and F3.

**Figure 2:**
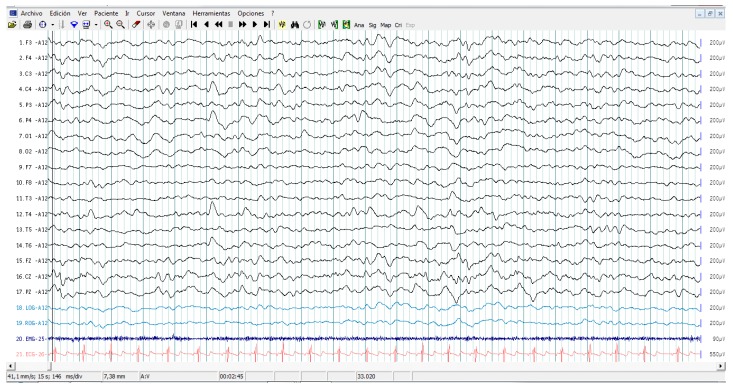
Sleep electroencephalography study in a patient with ASD without epileptic seizures. Recordings involve complete electroencephalogram, chin electromyography, eye movements, and electrocardiogram. Note EEG epileptiform abnormality in channels containing the right centro parieto-temporal leads, C4, P4, T4, and T6.

All patients showed interictal epileptiform activity with EEG, however, 37.5% of them had epilepsy. The abnormalities with EEG were focused on brain frontal-temporal region in 50% of the autistic patients tested, but it was not significantly associated with epilepsy (Fisher exact test *p* = 0.58). Figure 1 and Figure 2 represent the EEG measures in autistic patients with and without epileptic seizures.

### 3.3. Cytokine Profile Level in ASD

**Figure 3:**
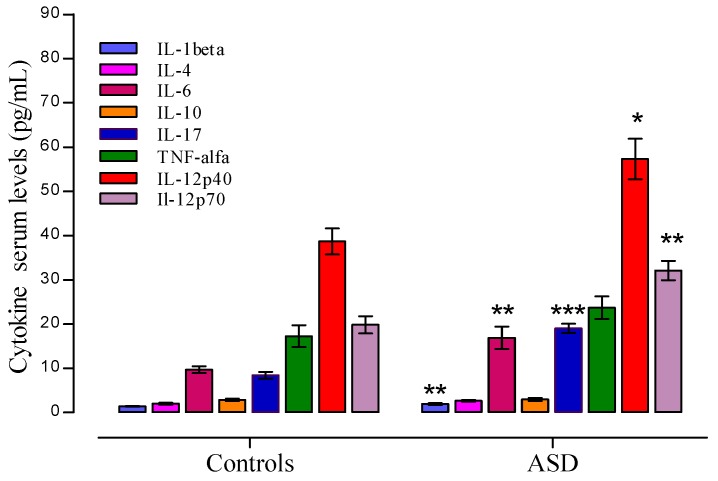
Plasma cytokine levels in Autism spectrum disorders (ASD) patients and controls. Significant data are showed as median ±SEM. Significant levels to * *p* < 0.05, ** *p* < 0.01, and *** *p* < 0.001, *t* Student test.

Plasma levels of IL-4, IL-7, IL-10, and TNF-α, IL-12p40, and IFNγ were higher in the ASD group than those observed in AMTD, but they did not reach statistical significance after correction for multiple comparisons. Student *t* test rich differences of cytokines levels were as follow: IL-1β (*t* = 3.245, *p* = 0.0028), IL-6 (*t* = 3.439, *p* = 0.0017), IL-17 (*t* = 7.712, *p* = 0.0000), IL-12p40 (*t* = 2.917, *p* = 0.0065), and IL-12p70 (*t* = 3.758, *p* = 0.0007), in the autistic group compared with the corresponding values of the AMTD group. Figure 3 shows the median values of cytokines that differed between the ASD patient group and the control group. Values are given as median ± SEM. 

### 3.4. Cytokine Profile and Behavioral Clinical Outcomes in Autistic Group

#### Cytokine Profile and Disease Severity in Autistic Patients

Autistic patients were divided into two subgroups: patients with moderate (PMDS) and mild (PmDS) severity. Autistic patients with severe state of the disease were excluded from the analysis (*n* = 1). Differences in respect to disease severity were observed in IL-1β, IL-6, TNFα, IL-17, IL-12p40 and IL-12p70.

**Figure 4:**
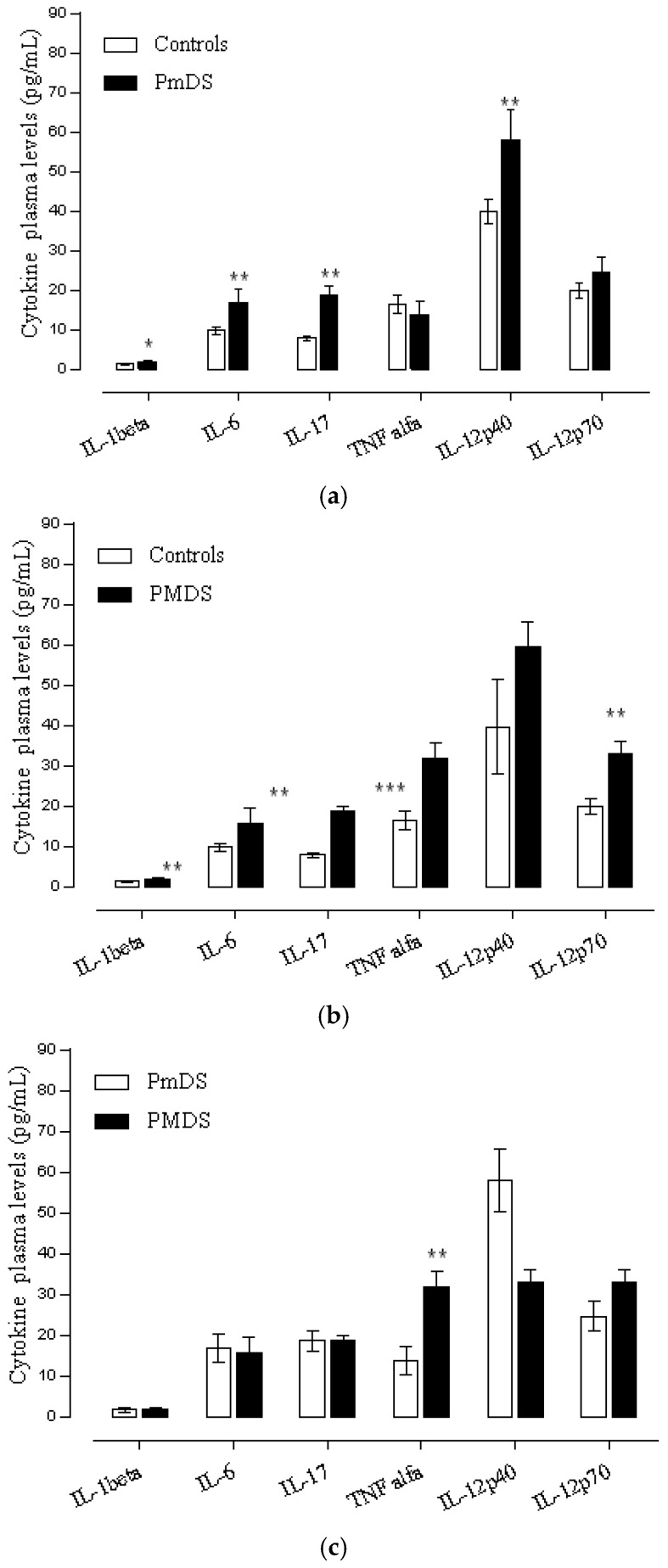
(**a**) Plasma cytokines level in autistic patients with mild disease severity and controls. Data are shown as median ±SEM. Significant differences, * *p* < 0.05, ** *p* < 0.01. *t*-Student test, PmDS (Mild disease severity); (**b**) Plasma cytokines level in autistic patients with moderate disease severity and controls. Data are shown as median ± SEM. Significant differences, * *p* < 0.05, ** *p* < 0.01, *** *p* < 0.001. *t*-Student test, PMDS (Moderate disease severity); (**c**) Plasma cytokines level in autistic patients with mild and moderate disease severity. Data are shown as median ± SEM. Significant differences, * *p* < 0.05, ** *p* < 0.01. *t*-Student test, (PmDS vs. PMDS): Mild vs. Moderate disease severity.

The comparison of patients with different degrees of severity with respect to the control group shows differences as follow: autistic patients with mild disease severity showed significant differences in IL-1β (*t* = 2.61, *p* = 0.017), IL-6 (*t* = 3.68, *p* = 0.0015), IL-17(*t* = 4.45, *p* = 0.0002), and IL-12p40 (*t* = 3.27, *p* = 0.0039) (see Figure 4a), while autistic patients with moderate disease severity showed significant differences in IL-1β (*t* = 3.01, *p* = 0.006), IL-6 (*t* = 3.48, *p* = 0.0022), IL-17 (*t* = 7.69, *p* = 0.000), TNFα (*t* = 2.61, *p* = 0.0162), and IL-12p70 (*t* = 4.22, *p* = 0.0004) (see Figure 4b).

The comparison between the patients with mild disease severity (mDS) and moderate disease severity (MDS) showed that there were higher levels of IL-12p40 in patients with mild disease severity and higher levels of IL-6, TNFα, and IL-12p70 cytokine in patients with moderate severity of the disease. Nevertheless, the *t* Student test shows significant differences only in TNFα when comparing both groups. IL-1β levels were similar in both groups (see Figure 4c).

### 3.5. Cytokine Profile and EEG Findings in Autistic Patients

Following the EEG measures showed in this analysis, patients were classified as either individuals with history of epilepsy or without history of epilepsy.Higher median values of IL-12 p40 was found in autistic patients with history of epilepsy compared to those without epilepsy (*z* = 2.23, *p* = 0.025). On the other hand, IL-6 levels were higher in patients without epilepsy (*z* = 2.30, *p* = 0.021), underlined by an interictal epileptiform activity focused in the frontal brain region.

## 4. Discussion

Cytokines are molecules with autocrine, paracrine, and endocrine functions which act to regulate the cross talk between the immune and nervous system via receptors expressed in neural and immune cells. In this context, precursors of mature neuronal cells became specialized to modulate the neurogenesis processes at time that can be influenced to induce an aberrant maturation, proliferation, and survival of neuron precursor cells [3,25].

Despite the dogma that peripheral immune responses could not affect central nervous system (CNS) function under normal circumstances, substantial evidence over the past 10 years suggests that immune-CNS cross-talk may be the norm rather than the exception. Thus, peripheral immune cells can alter cognition in the absence of CNS immune cell infiltration, suggesting that neural-immune cross-talk may involve more than the simple “breaching” of the blood brain barrier (BBB). Multiple members of the large family of cytokines are normally produced in the healthy brain where they play critical roles in almost every aspect of neural development, including neurogenesis, migration, differentiation, synapse formation, plasticity, and responses to injury [26,27]. As such, the early cytokine alteration influencing the brain function may identify the onset of developmental disorders like autism as well as underline a particular pattern of disease progression or severity [28,29,30].

Multiples papers reporting immune changes in autism were based on cytokine analysis [31,32,33,34,35,36,37]. In the last five years, more than 700 published articles have reported differential patterns of cytokine and other mediators in fluids and brain tissue of individuals with ASD (data taken from PubMed Central, August 2016), nevertheless there has not been a consensus on the contribution of cytokine to clinical behavioral outcome and comorbidities [38,39].

Changes to plasma cytokine levels in individuals with ASD have provided evidence of immune dysfunction associated with or without an impaired behavioral outcome. Indeed, Ashwood and colleagues found significant increases of IL-1β, IL-6, IL-8, and IL-12p40 in the plasma of autistic patients predominantly showing a severe disease form [31], whereas other research groups reported no association between clinical assessment and cytokine profile in autism [40,41,42,43].

IL-1β plays a key role mediating severe placental damage and neurodevelopmental anomalies in offspring [44]. From this point of view, our results reinforce its potential involvement in the physiopathology of autism. Evidence of variations in plasma IL-1β levels in children with ASD have been shown: (1) higher levels were found in high-functioning children with ASD and adults with severe ASD compared with unrelated controls [45]; (2) higher levels of IL-1β were shown to have been released both at baseline and after stimulation with Toll like receptor (TLR)2 or TLR4 [45] from peripheral blood cells from subjects with ASD [31]; and (3) increased cytokine production of IL-1β levels were found in children with regressive forms of ASD. Other published studies also have considered high levels of IL-1β in fetal brains suggesting that IL-1β may have a deleterious impact on the development of the central nervous system [44,46].

The IL-17 is a pro-inflammatory cytokine that is secreted by a specific T-cell subset termed Th-17 in response to inflammatory stimulus and has been identified as the major regulator of CNS autoimmunity. This cytokine induces the release of IL-6 and synergizes with IL-1β to mediate the autoimmune mechanism [47]. In this line of findings, the peripheral upregulation of IL-17 and IL-1β observed in this study could suggest their role in the neurotoxicity reported in autism, supported by an autoimmune mechanism, similar to those reported in other neurodegenerative disorders like multiple sclerosis [48]. Specific associated EEG patterns are used to classify different epilepsy syndromes. So, regional electrographic abnormalities in concurrence with molecular changes could be useful for establishing major and earlier precision of diagnosis of the disease, thereby likely having a favorable impact on treatment and prognosis.

Autism is characterized by a complex interaction of genetic, environmental, and immunological factors. The IL-1/IL-17 axis exhibits a pro-inflammatory effect on immune and neural cells, inducing the differentiation of naïve CD4 T cells via IL-1, and promoting their differentiation to naïve Th17 cells with the secretion of IL-17 cytokines. So, in the context of an up-regulated pro-inflammatory response, the aberrant chronic inflammatory response in autism can be amplified with negative effects on the brain. It is well known that IL-17 acting on inflammatory brain cells induces major secretion of IL-1β, TNFα, and MMP9 which causes neurotoxicity and neuronal death by an apoptotic mechanism. Increased levels of IL-1 and IL-17 observed in autism support this hypothesis. At the same time, the measures of TNFα in patients with moderate disease severity suggest apoptosis during a critical stage of neurodevelopment.

Many children and adults with a diagnosis of ASD have comorbid health problems. Recent large-scale studies have confirmed that several medical conditions are significantly more prevalent in people with autism compared to the typical population [49,50,51]. It has been suggested that these conditions may be markers for underlying pathology and require a more varied treatment approach.

Epilepsy and autism, for example, are common comorbidities [50,52], although besides the prevalent correlation between both disorders, there are not enough reports contributing to clarify the molecular mechanism involved. Behavioral differences have been reported inautistic patients displaying different EEG patterns [53,54]. In this paper, differential changes in IL-6 and IL-12p40 related to EEG findings, thereby suggesting that a down-regulated expression of IL-6 in combination with an up-regulated IL-12p40 could be an element related to a risk of epilepsy comorbidity in autism.

A pathological increase in IL-6 secretion can be the result of an up-regulation of IL-1β in individuals with autism [55] at the time that IL-1β also acts to induce the expression of transcription factors, such as NFkB, which also somewhat controls the expression of genes encoding other cytokines, such as IL-6 and INFγ. In this context, changes observed in this paper to IL-6 could be considered not only to support considerations surrounding neuroinflammation in autism, but also to supportits potentialas a peripheral marker in the interpretation of clinical findings related to comorbidities in autism, with the particular suggestion of considering the frontal localization of epileptic focus, since the frontal pathology is prominent in autism [56]. Changes in IL-6 levels could contribute to the understanding of the molecular phenotype associated with the expression of varying degrees of ASD comorbidity.These data support a model of molecular evidence linking phenotype and endophenotype associated with immune system dysregulation, and supporting cytoarchitectural changes which may be mediated by immune response [56,57,58]. The cerebral cortex provides the mainboard of critical circuitry that ensure the emergence of higher cognitive functions such as language, memory, and attention, among others. Bailey and colleagues were pioneering studies emphasizing the role of cerebral cortex in the neuropathology of ASD [59]. Nevertheless, it is well known that an impairments of corticogenesis themselves induce a neurodevelopmental disorder, manifested by deficits in neuronal migration that also promote excitatory/inhibitory bias capable of explaining the occurrence of comorbid seizure disorder.

The interpretation of many of the clinical findings in ASD appears related to the minicolummnar pathology and the inhibitory defect in the cerebral cortex in these individuals focused around its minicolumnar organization [56,60]. So, the inhibitory deficit procreated by the minicolumnopathy, offers a suitable relationship with the seizures observed in many autistic patients [60,61]. Following this line of thought, a maldeveloped cytoarchitecture within the prefrontal cortex, in a critical period of postnatal development, could be related to a dysregulated autoimmune reaction, which may result in neurotoxicity and neuronal death, thus impacting neural connectivity or inducing atypicalneural connectivity as measured by EEG.

Differences have been reported by other studies, like those published by Saresella and Napoleoni [62,63], which referred to the influence of environmental stimulus acting to induce neurotoxicity. This process could affect the natural “pruning process” of apoptosis, which is essentially necessary to the formation of optimal neural pathways during the early stages of typical development, a feature to consider for future research projects. The great complexity of abnormal brain architecture, the dysregulated synaptic plasticity and the aberrant heterogeneity of the immune reaction occurring in this disorder [31,62,63] are main elements to explore to depth of this pathology. Nevertheless, clinical protocols continue to be necessary to obtain major comprehension on the altered neural plasticity and behavioral features of these patients.

A prolonged local inflammatory stimulus has been underlined as a vulnerable factor which may induce neuronal toxicity, hyperexcitability, and higher susceptibility to damage. The changes in peripheral cytokines observed in this study in relationship to clinical and paraclinical parameters support the hypothesis on an impaired neuroplasticity occurring in autism [12,13,14,15,16,34,64,65].

A weakness of this preliminary work could be considered from the point of view of the age range of the patients, since language development changes drastically between the age of three and nine years. That is why we suggest continuing onwards with this analysis presented here, with impact of ASD on language, and others measures demonstrating sensitivity to factors based on developmental level and chronological age suggested for further consideration in future research in order to exclude from the analysis factors based on developmental level and chronological age with impact on disease severity.

Finally, this article explored the role of cytokines in the expression of ASD, from a different clinical view point, namely, their influence on the expression of endophenotype by severity, in line with measures of EEG. Although there is still a debate in the ASD literature, the preliminary evidence of this paper suggests an interesting relationship for future studies involving ASD severity and comorbid seizure disorder. The measure of Intelligence Quotient or verbal vs. nonverbal communication could also represent helpful areas of consideration to be explored in future studies. The results presented in this paper relating EEG results and ASD severity with immune system dysregulation and the presence of cytokines as well as the identification of “potential biomarkers” represents an interesting discovery to be explored with further depth in future studies.

## 5. Conclusions

This article presents a preliminary finding regarding the role of cytokines on the expression of ASD, describing the molecular process of immune response and also the role of cytokines (biomarkers) on the expression of endophenotype by severity, in line with measures of EEG. Although still a debate in the ASD literature, the mechanism by which the immune response is described here suggests an interesting relationship and could inform future studies examining ASD severity and comorbid seizure disorder. The finding relating brain function to immune response further supports existing evidence that peripheral toxins cross the blood brain barrier and may have a more significant contribution to the expression of ASD as a result of neurotoxicity and dysregulated immune response during a critical period of neural development. This process could affect the natural “pruning process” of apoptosis, which is considered essential for forming efficient neural pathways during the early stages of typical development. Additionally, other measures demonstrating sensitivity to factors based on developmental level and chronological age are suggested for future studies in order to exclude factors based on developmental level and chronological age with impact on disease severity. Other studies must also consider clinical measure like verbal vs. nonverbal communication, which might be helpful to confirm evidence presented in this paper.

At the same time, the aberrant connectivity (see above) also shows an impact on the expression of behavioral patterns of ASD in line with comorbid seizure disorder. In this case, other studies measuring EEG predictive validity with ASD diagnosis and severity according to a more clearly defined standard of clinical phenotype and endophenotype would be useful to reduce probable confounds of heterogeneity within the individuals with ASD studied.

## Figures and Tables

**Table 1:** Clinical and demographic characteristics of autistic patients. CARS, Childhood Autism Rating Scale; M, male; F, female.

Patient	Age (Years)	Sex	CARS Score	Severity
1	9	M	26	Mild
2	7.8	M	35.5	Moderate
3	7.8	M	36.5	Moderate
4	5	M	33	Moderate
5	6.1	M	36	Moderate
6	4	F	46	Severe
7	6.6	F	33.5	Moderate
8	4	M	36	Moderate
9	9.1	M	28	Mild
10	8	M	37	Moderate
11	5.2	F	36.5	Moderate
12	3	M	35.5	Moderate
13	3	F	27	Mild
14	7.2	M	34.5	Moderate
15	9.1	M	35	Moderate
16	5	F	30	Mild
17	5	M	31	Mild
